# Psychiatric Health Risks in North Korean Refugee Youths Resettled in South Korea

**DOI:** 10.1001/jamanetworkopen.2025.12941

**Published:** 2025-05-29

**Authors:** Rugyeom Lee, Sang Min Lee, Minha Hong, In-Hwan Oh

**Affiliations:** 1 Department of Preventive Medicine, Kyung Hee University School of Medicine, Seoul, South Korea; 2 Department of Psychiatry, Kyung Hee University Hospital, Kyung Hee University College of Medicine, Seoul, South Korea; 3 Department of Psychiatry, Kyung Hee University Hospital at Gangdong, Kyung Hee University College of Medicine, Seoul, South Korea; 4 Department of Preventive Medicine, University of Ulsan College of Medicine, Seoul, South Korea

## Abstract

**Importance:**

Migrant and refugee populations are increasing globally, and children and adolescents in these populations are particularly susceptible to mental disorders. North Korean refugee (NKR) youths now living in South Korea share a culture, language, and history with South Korean (SK) youths, making these 2 groups suitable for studying the environmental factors in psychiatric health.

**Objective:**

To compare the risk of developing mental illness and individual psychiatric disorders among NKR youths and SK youths.

**Design, Setting, and Participants:**

This cohort study obtained data from Korea’s National Health Insurance Service claims database from 2005 to 2021. Participants were aged 1 to 18 years between 2007 and 2010 who had no prior psychiatric claims, whether as outpatients or inpatients. Children and adolescents of the SK general population matched 1:10 on sex and age were compared with the NKR youth population. Statistical analyses were performed from August 2024 to March 2025.

**Main Outcomes and Measures:**

Risk, presented as hazard ratio (HR), of developing psychiatric disorders in NKR youths compared with SK youths. This risk was assessed using a Cox proportional hazards regression model and Kaplan-Meier time-to-event probabilities.

**Results:**

In total, 1618 NKR youths (810 males [50.1%]; mean [SD] age, 9.48 [4.62] years) and 308 927 SK youths (194 331 females [62.9%]; mean [SD] age, 11.80 [4.72] years) were included in this study. NKR youths showed a significantly higher risk of developing psychiatric disorders (HR, 1.29; 95% CI, 1.17-1.43) compared with SK youths. Individual psychiatric disorder risks were higher for NKR youths, including posttraumatic stress disorder (HR, 2.33; 95% CI, 1.34-4.06; *P* = .003), attention-deficit/hyperactivity disorder (HR, 1.67; 95% CI, 1.32-2.11; *P* < .001), bipolar affective disorders (HR, 1.61; 95% CI, 1.20-2.15; *P* < .001), major depressive disorder (HR, 1.53; 95% CI, 1.33-1.75; *P* < .001), and anxiety (HR, 1.25; 95% CI, 1.11-1.42; *P* < .001).

**Conclusions and Relevance:**

In this cohort study, compared with SK youths, NKR youths showed a significantly higher risk of developing psychiatric disorders overall and individual psychiatric disorders, such as posttraumatic stress disorder, attention-deficit/hyperactivity disorder, bipolar disorder, major depressive disorder, and anxiety. This finding is important for establishing medical and educational service plans and policies for the increasing number of refugee youths in South Korea.

## Key Points

**Question:**

Is there a difference in the risk of mental disorders between North Korean refugee children and adolescents who have settled in South Korea and South Korean children and adolescents?

**Findings:**

In this cohort study of 1618 North Korean youths and 308 927 South Korean youths, North Korean refugee children and adolescents had a higher risk of developing mental illness and individual psychiatric disorders than South Korean children and adolescents.

**Meaning:**

This finding is important for establishing medical and educational service plans and policies for the refugee youth population in South Korea.

## Teaser

This cohort study assesses the incidence and risk of mental disorders among North Korean refugee youth and general populations of children and adolescents in South Korea.

## Introduction

On August 15, 1945, Japan surrendered, ending World War II. After the Japan-Korea Annexation Treaty, the Korean Peninsula, which had been under forced Japanese rule, was divided into South and North Korea along the 38th parallel, with US and Soviet troops stationed in the South and North, respectively. On July 27, 1953, a ceasefire was established to fix the division. The year 2015 marked the 70th anniversary of the liberation and division of Korea, 2018 marked the 70th anniversary of the establishment of the North and South governments, 2020 marked the 70th anniversary of the Korean War, and 2023 marked the 70th anniversary of the armistice.

Currently, the Korean Peninsula is the only divided monocultural region. For more than half a century, Korean people, sharing a culture, language, and history, have been separated by different political systems. Each year, a substantial number of North Koreans enter South Korea via various channels. Although the annual influx decreased during the COVID-19 pandemic of 2020 to 2022, it has increased at a rate of more than 1000 people per year since 2000, reaching a cumulative total of 34 000 by the end of 2023.^1^ Those who leave North Korea and settle in South Korea receive support and education from the South Korean (SK) government for a certain period, and medical insurance is managed separately.

Previous studies have reported poorer mental health outcomes for North Korean refugee (NKR) youths compared with SK youths.^2,3^ NKR youths have a higher incidence of depression, anxiety, and suicidal ideation; more visits to psychiatric clinics; and higher rates of major depressive disorder (MDD) and posttraumatic stress disorder (PTSD).^2,4^ A study compared 102 participants from a public educational institution for NKR adolescents in South Korea with 766 adolescents from the same area using the Korean version of the Child Behavior Checklist and found that NKR adolescents had severe internalization problems.^4^

The extant literature on migrant and refugee populations has limitations, such as lack of a control group and a small sample size (including only a few regions). Nevertheless, depression, anxiety, and sleep disturbances, often in combination, have been reported to be more common among refugee children than among the general population.^5,6,7^

Analyzing these groups in monocultures makes sense for several reasons. As they share the same language (written and spoken), which is a large part of human culture, they have different characteristics from other refugee groups, and differences in their migration environments are likely to be the main focus. Children and adolescents are still developing their brains; therefore, it is possible to identify the role of environmental factors in their psychiatric health.

In this study, we aimed to compare the risk of developing mental illness and individual psychiatric disorders among NKR youths and SK youths. Additionally, we compared the incidence of mental illness between the 2 groups according to the length of time lived in South Korea.

## Methods

The Kyung Hee University Institutional Review Board deemed this cohort study exempt from ethics review and the informed consent requirement because it followed the Korean guidelines for the deidentification of personal data. This study followed the Strengthening the Reporting of Observational Studies in Epidemiology (STROBE) reporting guideline.

### Data Source and Study Population

We used the Korea National Health Insurance Service (NHIS) claims database from 2005 to 2021, obtained the entire dataset of NKR population that could be verified by the NHIS, and compared the occurrence of mental illness in the SK general population aged 1 to 18 years and in NKR youths. The data included demographic information, such as sex, age, and insurance premium level as well as information on medical use, including disability. The control group (SK general population) was matched 1:10 by sex and age to the NKR youth, and only those aged 1 to 18 years were included in this study.

Children and adolescents aged 1 to 18 years from 2007 to 2010 who did not have a history of outpatient or inpatient medical service use due to mental illness in the 2 years (washout period) prior to the index point were included in the cohort using NHIS claims database from 2005 to 2021. Those who died or were diagnosed with mental illness during the washout period were excluded. The onset of mental illness was monitored until December 31, 2021. The maximum age for the observation period in which to confirm the onset of mental illness was 32 years.

### Statistical Analysis

Demographic variables are presented as mean (SD) and count (frequency) based on the initiation of observation. The occurrence of mental illness was determined by use of the *International Statistical Classification of Diseases and Related Health Problems, Tenth Revision* (*ICD-10*) F codes, based on hospitalizations or outpatient visits. Risk is presented as a hazard ratio (HR) calculated with the Cox proportional hazards regression model. Since the proportional hazards assumption was not satisfied, time-stratified analyses were performed by sex, age group, and follow-up period. The model was adjusted for sex, age, disability, and insurance premium level. Age was divided into groups for analysis: 1 to 5 years (preschool age), 6 to 12 years (school age), and 13 to 18 years (adolescence). Insurance premium level was divided into low, middle, and high, according to the 21 income-based contribution quantiles provided by the NHIS.

Two-sided *P* < .05 indicated statistical significance. Statistical analyses were performed from August 2024 to March 2025 using the SAS Enterprise Guide tool (SAS Institute Inc) provided by the NHIS.

## Results

The final sample included 1618 NKR youths (810 males [50.1%] and 808 females [49.9%]; mean [SD] age, 9.48 [4.62] years) and 308 927 SK youths (114 596 males [37.1%] and 194 331 females [62.9%]; mean [SD] age, 11.80 [4.72] years). The results are presented by age groups, including the overall results. Among NKR youths, 415 (25.6%) were aged 1 to 5 years, 754 (46.6%) were aged 6 to 12 years, and 449 (27.8%) were aged 13 to 18 years. SK youths comprised 40 446 (13.1%) aged 1 to 5 years, 105 175 (34.0%) aged 6 to 12 years, and 163 306 (52.9%) aged 13 to 18 years (Table 1).

**Table 1.** Baseline Characteristics of the Study Participants

Characteristic	Participants, No. (%)		*P* value
	SK youth (n = 308 927)	NKR youth (n = 1618)	
Age group, y			
1-5	40 446 (13.1)	415 (25.6)	<.001
6-12	105 175 (34.0)	754 (46.6)	
13-18	163 306 (52.9)	449 (27.8)	
Age continuous, mean (SD), y	11.80 (4.72)	9.48 (4.62)	<.001
Sex			
Male	114 596 (37.1)	810 (50.1)	<.001
Female	194 331 (62.9)	808 (49.9)	
Insurance premium level			
Low (0-6th)	64 727 (21.0)	1507 (93.1)	<.001
Middle (7th-13th)	90 989 (29.5)	92 (5.7)	
High (14th-20th)	153 211 (49.6)	19 (1.2)	
Disability			
Yes	1692 (0.5)	5 (0.3)	.19
No	307 235 (99.5)	1613 (99.7)	

Abbreviations: NKR, North Korean refugee; SK, South Korean.

### Risk of Mental Illness 

The incidence and risk of mental illness are presented in Table 2. The difference in developing mental illness between the groups was statistically significant. After 15 years of follow-up, the event-free rate in NKR youths decreased to less than 70%, indicating a cumulative increase in psychiatric morbidity over time. The Figure shows that the association of migration environment with mental health (measured by cumulative event-free probability) becomes more pronounced as time progresses. The incidence of patients with mental illness was 389 (or 2148.2 per 100 000 person-years) in NKR youths and 82 434 (or 2006.3 per 100 000 person-years) in SK youths. NKR youths had a higher incidence rate. The total observation period was 4 127 821.5 person-years, with a mean (SD) observation period of 13.3 (3.6) years for SK youths and 11.2 (3.4) years for NKR youths.

**Table 2.** Incidence and Risk of Psychiatric Morbidity by Age Group

Variable	No. of patients	Incidence, No.	Incidence per 100 000 person-years	HR (95% CI)^a^	*P* value
Total					
SK youth	308 927	82 434	2006.3	1 [Reference]	NA
NKR youth	1618	389	2148.2	1.29 (1.17-1.43)	<.001
Male sex					
SK youth	114 596	24 359	1569.4	1 [Reference]	NA
NKR youth	810	173	1895.5	1.32 (1.14-1.54)	<.001
Female sex					
SK youth	194 331	57 075	2238.9	1 [Reference]	NA
NKR youth	808	216	2404.9	1.30 (1.14-1.49)	<.001
Age 1-5 y					
SK youth	40 446	6575	1152.9	1 [Reference]	NA
NKR youth	415	82	1619.6	1.40 (1.12-1.75)	.003
Age 6-12 y					
SK youth	105 175	24 598	1719.7	1 [Reference]	NA
NKR youth	754	177	2114.8	1.32 (1.14-1.53)	<.001
Age 13-18 y					
SK youth	163 306	50 261	2404.5	1 [Reference]	NA
NKR youth	449	130	2811.0	1.27 (1.07-1.50)	.008

Abbreviations: HR, hazard ratio; NA, not applicable; NKR, North Korean refugee; SK, South Korean.

^a^ Multivariate Cox proportional hazards regression models were adjusted for age (continuous), sex, insurance premium level (low, middle, or high), and presence of disability.

**Figure.**
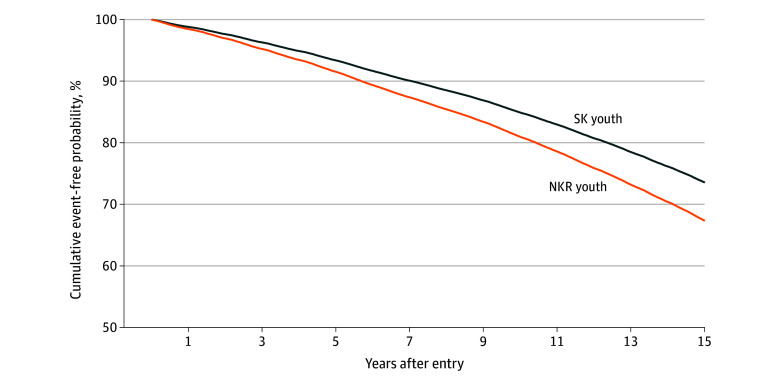
Event-Free Probability of Psychiatric Morbidity NKR indicates North Korean refugee; SK, South Korean.

When adjusting for sex, age, insurance premium level, and disability based on the overall age of the patients, the HR for mental illness in NKR youths was 1.29 (95% CI, 1.17-1.43) higher than in SK youths, which was statistically significant (*P* < .001). When stratified by sex, the risk of mental illness was significantly higher in both male and female NKR youths (HR, 1.32 [95% CI, 1.14-1.54] and 1.30 [95% CI, 1.14-1.49]; *P* < .001) compared with male and female SK youths. When stratified by age group, the HRs for mental illness among NKR youths were significantly high in all age groups: 1.40 (95% CI, 1.12-1.75; *P* = .003) for those aged 1 to 5 years, 1.32 (95% CI, 1.14-1.53; *P* < .001) for those aged 6 to 12 years, and 1.27 (95% CI, 1.07-1.50; *P* = .008) for those aged 13 to 18 years (Table 2).

In conducting additional time-stratified analyses, we divided follow-up time into early (0-2 years), intermediate (2-7 years), late (7-10 years), and extended (>10 years) periods. The HR for mental illness in NKR youths compared with SK youths was significantly elevated during the early (HR, 1.58; 95% CI, 1.20-2.08; *P* = .001), late (HR, 1.66; 95% CI, 1.38-1.99; *P* < .001), and extended (HR, 1.29; 95% CI, 1.07-1.57; *P* = .008) follow-up periods. In sex-stratified analyses, the increased risk was significant among males in both the early (HR, 1.67; 95% CI, 1.14-2.43; *P* = .008) and late (HR, 1.95; 95% CI, 1.49-2.55; *P* < .001) follow-up periods. Stratified by age groups, a significantly elevated risk was observed in children aged 6 to 12 years during the late follow-up period (HR, 1.60; 95% CI, 1.23-2.07; *P* = .001). Full results of the time-stratified analyses are provided in eTable 2 in Supplement 1.

The sex- and age-stratified results are presented in eTable 1 in Supplement 1. In this analysis, the risk of mental illness in NKR youths compared with SK youths was significantly elevated in males aged 13 to 18 years (HR, 1.45; 95% CI, 1.12-1.88) and in females aged 1 to 5 years (HR, 1.54; 95% CI, 1.14-2.10) and 6 to 12 years (HR, 1.42; 95% CI, 1.16-1.73).

### Risk of Individual Psychiatric Disorders

The risks associated with individual mental disorders are presented in Table 3. The risk for NKR youths compared with SK youths was significantly higher for PTSD (HR, 2.33; 95% CI, 1.34-4.06; *P* = .003), for attention-deficit/hyperactivity disorder (ADHD) (HR, 1.67; 95% CI, 1.32-2.11; *P* < .001), for bipolar affective disorders (HR, 1.61; 95% CI, 1.20-2.15; *P* < .001), for MDD (HR, 1.53; 95% CI, 1.33-1.75; *P* < .001), and for anxiety (HR, 1.25; 95% CI, 1.11-1.42; *P* < .001).

**Table 3.** Subanalysis for Risk of Individual Psychiatric Disorders

Disorder	Incidence, No.		HR (95% CI)^a^	*P* value
	SK youth [Reference]	NKR youth		
MDD	40 445	206	1.53 (1.33-1.75)	<.001
Bipolar affective disorder	8688	47	1.61 (1.20-2.15)	<.001
Anxiety, panic	59 858	253	1.25 (1.11-1.42)	<.001
PTSD	1223	13	2.33 (1.34-4.06)	.003
ADHD	7923	72	1.67 (1.32-2.11)	<.001

Abbreviations: ADHD, attention-deficit/hyperactivity disorder; HR, hazard ratio; MDD, major depressive disorder; NKR, North Korean refugee; PTSD, posttraumatic stress disorder; SK, South Korean.

^a^ Multivariate Cox proportional hazards regression models were adjusted for age (continuous), sex, insurance premium level (low, middle, or high), and presence of disability.

## Discussion

This nationwide, population-based cohort study found that NKR youths had a significantly higher risk of developing mental illness than SK youths. This finding was consistent even years after refugee migration to South Korea (Figure).

A retrospective, population-based cohort study compared the prevalence of conduct disorder, ADHD, and mood or anxiety disorders among immigrant, refugee, and nonimmigrant children and adolescents in British Columbia, Canada, using linked health administrative records.^8^ The study included 470 464 children and adolescents and reported that youths from immigrant and refugee backgrounds (first- and second-generation) had a significantly lower diagnosis of conduct disorder, ADHD, and mood or anxiety disorders. The result of this Canadian study is inconsistent with our findings. However, in the Canadian study, the authors cited barriers to accessing services (such as language skills and differences in treatment-seeking, including lower use of health services by immigrant groups from East Asia) as reasons for the lower prevalence of diagnosed mental disorders among first- and second-generation immigrant and refugee youths. These barriers can be assumed to be almost equally balanced in the present study and can be interpreted as a result of comparing refugees with nonimmigrants. Therefore, if there are no language barriers and fewer barriers to accessing health care (eg, high cost), the mental health risk in children and adolescents can be explained as higher in the refugee population.

We expected that, over time, the risk of mental disorders among NKR youths would decrease after resettlement to the level of the SK youths (due to factors such as better social environment and greater access to medical care in South Korea compared with North Korea), but we found that significant differences persisted over time (Figure). Hypothetical explanations include the following: environmental factors were not sufficiently large to offset the differences in prevalence, early adverse experiences during the migration process had a greater role in risk, and innate predisposition had a greater impact than environmental factors. Nevertheless, scientific verification of these results is required. Fazel and Betancourt^9^ discussed cultural differences that affect the role of research and the notion of mental illness as a barrier to conducting high-quality research.

### Strengths and Limitations

The strengths of this study include its large size and low heterogeneity of the sample, as it was a monocultural population with a shared history, culture, and language and included almost all NKR youths who have resettled in South Korea. Furthermore, although there are some published data on NKR adults,^3,10,11^ this is the first study, to our knowledge, to compare the risk of individual mental disorders in NKR child and adolescent populations. This study avoids premature conclusions about the potential reasons for the differences in mental health risk, which helps prevent common biases and promotes a more balanced interpretation of the findings.

### Limitations

Despite these strengths, the study has several limitations. Given that the NHIS is a secondary database, it lacks information on parents, other family members, and clinical variables, such as psychological testing results and laboratory findings. Moreover, the claims data were based on *ICD-10* codes for administrative purposes, and the study could not use diagnoses from the *Diagnostic and Statistical Manual of Mental Disorders* (Fifth Edition). Third, due to restriction on the accessibility of dataset, additional analyses, such as performing more detailed diagnostic checks, were not feasible within the scope of this study. The results should be interpreted with caution given the limitation in the data. Fourth, given the unique sociocultural and geopolitical characteristics of the SK context, where refugees from North Korea are a specific subset of the refugee population, caution is needed when generalizing these results to other regions with different populations. Future research in other refugee settings is needed to assess whether similar patterns of psychiatric disorder risks are observed. Fifth, the relative difference in the number of NKR youths and SK youths is a substantial limitation. The sample size for the refugee youth group was considerably smaller, which may affect the power of the statistical analyses and the generalizability of the finding. Sixth, it was difficult to establish causal associations based on these findings.

## Conclusions

This study showed that NKR children and adolescents had a higher risk of mental illness than SK children and adolescents. This risk was also significantly higher for major psychiatric disorders. In addition, the risk difference in the incidence of mental illness among NKR youths remained steady over the 10-year period. This gap appeared to widen as time progressed. This study was the first, to our knowledge, to provide nationwide population-level estimates of mental disorder incidence and risk among NKR youths and SK youths. The results of this study are important in the field of refugee research in child and adolescent psychiatry, as we compared refugee with general populations who share a culture, history, and language. This information is especially important for establishing medical and educational service plans and policies for the increasing number of refugee youths in South Korea. Additionally, this information is expected to play a role in preparing the Korean Peninsula for the future unification era.

## Notes

Supplement 1. **eTable 1.** Incidence and Risk of Psychiatric Morbidity According to Sex and Age-Group **eTable 2.** Hazard Ratios for Psychiatric Illness Risks Among North Korean Refugee Youth Compared to General Youth by Follow-up Period, Sex, and Age Group

Supplement 2.
Data Sharing Statement
