# Supplementary material for: Psychiatric Health Risks in North Korean Refugee Youths Resettled in South Korea
Source: JAMA Netw Open. 2025 May 29;8(5):e2512941. doi: 10.1001/jamanetworkopen.2025.12941 (PMC12123469; doi:10.1001/jamanetworkopen.2025.12941)
Supplement: Supplement 1. — eTable 1. Incidence and Risk of Psychiatric Morbidity According to Sex and Age-Group eTable 2. Hazard Ratios for Psychiatric Illness Risks Among North Korean Refugee Youth Compared to General Youth by Follow-up Period, Sex, and Age Group [file jamanetwopen-e2512941-s001.pdf]

Supplemental Online Content

Lee R, Lee SM, Hong M, Oh IH. Psychiatric health risks in North Korean refugee youths resettled in South Korea. *JAMA Netw Open*. 2025;8(5):e2512941. doi:10.1001/jamanetworkopen.2025.12941

eTable 1. Incidence and Risk of Psychiatric Morbidity According to Sex and Age-Group

eTable 2. Hazard Ratios for Psychiatric Illness Risks Among North Korean Refugee Youth Compared to General Youth by Follow-up Period, Sex, and Age Group

This supplemental material has been provided by the authors to give readers additional information about their work.

© 2025 Lee R et al. *JAMA Network Open*

eTable 1. Incidence and risk of psychiatric morbidity according to sex and age-group

			N	Incidence	100000 per person- year	HR (95% CI)	P value
**Male**	Total	SK youth	114,596	24,359	1569.4	1 [Reference]	
		NKR youth	810	173	1983.4	1.32 (1·14–1·54)	<.001
	1–5 years	SK youth	19,417	3,161	1171.1	1 [Reference]	
		NKR youth	211	39	1540.1	1.27 (0·92–1·76)	.14
	6–12 years	SK youth	45,930	9,559	1527.7	1 [Reference]	
		NKR youth	375	76	1796.8	1.24 (0·99–1·56)	.06
	13–18 years	SK youth	49,249	11,639	1773.0	1 [Reference]	
		NKR youth	224	58	2452.5	1.45 (1·12–1·88)	.005
**Female**	total	SK youth	194,331	57,075	2238.9	1 [Reference]	
		NKR youth	808	216	2145.3	1.30 (1·14–1·49)	<.001
	1–5 years	SK youth	21,029	3,414	1138.2	1 [Reference]	
		NKR youth	204	43	1703.6	1.54 (1·14–2·10)	.006
	6–12 years	SK youth	59,245	15,039	1859.4	1 [Reference]	
		NKR youth	379	101	2414.7	1.42 (1·16–1·73)	<.001
	13–18 years	SK youth	114,057	38,622	2681.3	1 [Reference]	
		NKR youth	225	72	3165.1	1.17 (0·93–1·48)	.18

© 2025 Lee R et al. *JAMA Network Open*

eTable 2. Hazard Ratios for Psychiatric illness risks Among North Korean Refugee Youth Compared to General Youth by Follow-up Period, Sex, and Age Group

		Early period (0-2y)		Intermediate period (2-7y)		Late period(7-10y)		Extended period (10y <)	
		**HR (95% CI)**	**P value**	**HR (95% CI)**	**P value**	**HR (95% CI)**	**P value**	**HR (95% CI)**	**P value**
**Total**	SK youth	1 [Reference]		1 [Reference]		1 [Reference]		1 [Reference]	
	NKR youth	1.58 (1.20-2.08)	.001	1.00 (0.83-1.21)	.99	1.66 (1.38-1.99)	<.001	1.29 (1.07-1.57)	.008
**Male**	SK youth	1 [Reference]		1 [Reference]		1 [Reference]		1 [Reference]	
	NKR youth	1.66 (1.14-2.43)	.008	1.04 (0.80-1.37)	.75	1.95 (1.49-2.55)	<.001	1.04 (0.74-1.45)	.82
**Female**	SK youth	1 [Reference]		1 [Reference]		1 [Reference]		1 [Reference]	
	NKR youth	1.67 (1.11-2.50)	.01	1.02 (0.79-1.32)	.89	1.48 (1.15-1.90)	.002	1.49 (1.18-1.88)	.001
**1-5 years**	SK youth	1 [Reference]		1 [Reference]		1 [Reference]		1 [Reference]	
	NKR youth	1.82 (0.93-3.57)	.08	1.24 (0.82-1.88)	.30	1.62 (0.96-2.74)	.07	1.34 (0.95-1.89)	.09
**6-12 years**	SK youth	1 [Reference]		1 [Reference]		1 [Reference]		1 [Reference]	
	NKR youth	1.65 (1.09-2.49)	.02	0.94 (0.70-1.27)	.68	1.60 (1.23-2.07)	<.001	1.27 (0.96-1.68)	.09
**13-18 years**	SK youth	1 [Reference]		1 [Reference]		1 [Reference]		1 [Reference]	
	NKR youth	1.46 (0.93-2.30)	.10	0.96 (0.60-1.55)	.87	1.62 (1.20-2.18)	.002	1.29 (0.86-1.95)	.22

© 2025 Lee R et al. *JAMA Network Open*
