# Supplementary material for: Psychiatric Health Risks in North Korean Refugee Youths Resettled in South Korea
Source: JAMA Netw Open. 2025 May 29;8(5):e2512941. doi: 10.1001/jamanetworkopen.2025.12941 (PMC12123469; doi:10.1001/jamanetworkopen.2025.12941)
Supplement: Supplement 2. — Data Sharing Statement [file jamanetwopen-e2512941-s002.pdf]

Data Sharing Statement

Lee. Psychiatric Health Risks in North Korean Refugee Youths Resettled in South Korea. *JAMA Netw Open*. Published May 29, 2025. doi:10.1001/jamanetworkopen.2025.12941

Data
Data available: Yes
Data types: Other (please specify)
Additional Information: The dataset cannot be shared directly by the authors. However, access may be granted by the National Health Insurance Service (NHIS) upon request and institutional approval.
How to access data: Researchers may request access to the dataset through the NHIS data application system at: https://nhiss.nhis.or.kr Access is subject to NHIS review and institutional approval.
When available: Upon the policy and permission of NHIS

Supporting Documents
Document types: None

Additional Information
Who can access the data: Researchers whose proposals have been reviewed and approved by the NHIS under its data access policy.
Types of analyses: For research purposes consistent with NHIS data use guidelines and subject to NHIS approval.
Mechanisms of data availability: After approval of a formal data request and a signed data access agreement with NHIS.
Any additional restrictions: The data include sensitive information on North Korean refugee youth and cannot be publicly shared. All access is restricted and subject to NHIS institutional policies.
